# Global Disparities in Access to Vaccine Clinical Trials: A Review of the Literature

**DOI:** 10.3390/vaccines12040348

**Published:** 2024-03-23

**Authors:** Ali Mardini, Norhan Shaykhon, Asher Khan, Ahmad Mardini, Hajirah N. Saeed

**Affiliations:** 1 School of Medicine, Royal College of Surgeons in Ireland—Medical University of Bahrain, Busaiteen 228, Bahrain; 20200522@rcsi.com (A.M.); 18219683@rcsi.com (N.S.); asher.khan14@gmail.com (A.K.); 2 York Hospital, York and Scarborough Teaching Hospital and Foundation Trust, National Health Service (NHS), London SM5 1AA, UK; 3 Advocate Christ Medical Center, Chicago, IL 60453, USA; ahmad.mardini@aah.org; 4 Department of Ophthalmology, Illinois Eye and Ear Infirmary, University Illinois Chicago, Chicago, IL 60612, USA; 5 Department of Ophthalmology, Loyola University Medical Center, Maywood, IL 60153, USA; 6 Harvard Medical School, Boston, MA 02115, USA

**Keywords:** vaccine development, healthcare disparities, inequity, clinical trials

## Abstract

Vaccines are an effective tool to reduce the disease burden from infectious diseases on a population, infrastructural, and individual level. Before vaccines can be administered to populations at large, they must go through rigorous testing in the form of clinical trials. While vaccine trials can be used to assess the efficacy of interventions on a local populace as well as target local endemic diseases, most clinical trials are sponsored and conducted by companies in high-income countries (HICs). This can lead to vaccines that are not optimized for low- and middle-income countries (LMICs) and that often neglect to address diseases specific to the local population. This narrative review aims to explore the factors leading to discrepancies in the execution of and access to vaccine trials between HICs and LMICs, thus guiding future efforts in confronting them. This review was written using the literature sourced from the PubMed database and supplemented with articles from Google Scholar along with grey literature. Several themes are highlighted including poorly defined regulatory and ethical guidelines, staff shortages, lack of research infrastructure, and logistical barriers. We discuss how these challenges have affected vaccine development in various capacities through case examples of SARS-CoV-2, poliovirus, and malaria. Many challenges remain in equitable vaccine clinical trial development and implementation. Facilitating the implementation of locally sponsored vaccine clinical trials in LMICs may be one avenue to address these challenges. In doing so, LMICs can become active stakeholders in the health of their citizens by addressing endemic diseases, tailoring vaccine specifications based on local needs, and implementing wide-scale vaccine access and delivery.

## 1. Introduction

Clinical trials are critical for evidence generation and examination of novel interventions. Though they can be challenging in terms of study design, ethics, costs, and field implementation, they form the foundation for healthcare advancements, allowing the introduction of novel medical therapeutics embedded in the highest quality evidence [1]. Typically, they take the form of a study in which one cohort, the experimental arm, receives the new intervention and is compared against a control group that either receives a placebo or the standard care normally available [2].

Clinical trials are subjected to scrutiny at multiple levels by institutional review boards (IRBs) and federal agencies to ensure ethical conduct of research; namely, to protect the rights and welfare of research subjects. IRBs adhere to three guiding principles: respect for persons, beneficence, and justice. The latter includes ensuring burdens and benefits are shared equitably. Conclusions from clinical trials are most applicable to the populations which the study sample is representative of, and, in this way, the benefits of a trial are shared equitably in that population. Though IRBs may encourage adequate representation of the community in study recruitment, it is difficult to regulate and ensure; and inherent selection biases, where recruited research participants do not give a true reflection of the larger population in whom treatment is intended, do occur [3]. A systematic review of clinical trials in the English-language literature revealed that in more than 70% of 52 studied trials, significant disparities existed between the study sample and the general population of patients [4]. Another study of 230 US-based vaccine clinical trials showed many ethnic minority groups to be under-represented [5]. This disparity between study and general populations is further magnified on the international scale, where entire countries and regions of the world are under-represented in clinical trials of interventions intended for global use. Low- and middle-income countries (LMICs) bear the greatest global burden of disease, particularly infectious diseases. However, research, including clinical trials, on these diseases is lacking [6]. 

The COVID-19 pandemic put clinical trials and the race to vaccine development in the public eye. As countries around the globe directed research efforts to develop a vaccine, vaccination trial centers took the stage to recruit as large and diverse a population as possible. Vaccines against SARS-CoV-2 by Pfizer, AstraZeneca, and Sputnik V and others were tested internationally in high-income countries (HICs), middle-income countries (MICs), and low-income countries (LICs) alike, bolstering the external validity of trial results [7,8]. Though the race to develop a vaccine against SARS-CoV-2 highlighted the significance of collaboration and inclusivity in clinical trial sites and recruitment, it also underscored the significant barriers that still exist in equitable vaccine trial implementation and access.

Over the past several decades, there has been a global shift in clinical trial activities with clinical trials sponsored by the US and other Western HICs being conducted in LMICs [9]. While there are both benefits and detriments to this, the emergence of this trend raises many questions about why companies and organizations based in LMICs largely do not conduct clinical trials in their home countries, and the effect of globalization of vaccine clinical trials on local populations. 

We aim to investigate global trends in vaccine clinical trial implementation and access, and to identify barriers in conducting these trials in LMICs. 

## 2. Materials and Methods

A literature search was conducted primarily using results from PubMed, with limits set for English articles only and full-text availability. The search strategy is detailed in Appendix A. Following the initial search, articles were screened by title and abstract, after which the authors reviewed the full texts of relevant articles. The included articles were analyzed and underwent data extraction by four independent researchers. Any conflicts concerning the relevance of an article were settled by consensus.

The results were also supplemented with relevant articles from Google Scholar and the surveying of grey literature (sources are as described under Results) until data saturation was achieved. 

We used the World Bank’s definition of HICs for this review and interchangeably refer to such countries as “developed countries” depending on the source material. All other World Bank income classifications for countries less economically capable are referred to as LMICs or “developing countries”, depending on the source material [10,11].

*PubMed Search Strategy*: The search strategy involved utilizing MeSH terms for MeSH indexed articles: “Vaccines”, “Vaccine Development”, “Clinical Trials as Topic”, “Healthcare Services Accessibility”, “Healthcare Disparities”, “Low income countries”, “Middle income countries”, and “Developing Countries”. We also surveyed the literature for articles containing synonyms of these terms (obtained from the MeSH database) in the title and abstract. The resulting search had several iterations and revisions in order to capture the highest number of relevant articles. The exact search is included in Appendix A section at the end of this paper.

## 3. Results

Our PubMed search generated 135 articles of which 75 were included for full text review. In total, 17 out of the 75 papers were utilized for the synthesis of our findings. These papers included nine opinion pieces; two literature reviews, one of which was a systematic review; two cross-sectional studies; one cross-country analysis; one conference proceeding; one qualitative grounded theory study; and one short report. In addition to the PubMed articles, five webpages and five Google Scholar articles (one sounding board, one cross-sectional analysis, one editorial, and two perspective articles) were cited. We share the perspectives of these articles and provide insights and lessons learned from specific vaccine clinical trials.

### 3.1. Mapping Clinical Trial Settings

Developing countries account for approximately 90% of the world’s burden of disease, most of which consists of preventable infectious diseases [6]. Due to a shortage of resources and lack of commercial viability, these countries are under-represented in research. At the same time, the research interests and target goals of pharmaceutical companies are often driven by the needs of HICs. Consequently, diseases prevalent in HICs are studied via clinical trials up to eight times more than diseases prevalent in LMICs [6]. However, strict regulations on drug development in countries like the United States (US), and substantial potential cost savings, have resulted in a recent exodus of pharmaceutical-sponsored research from the US and other HICs to LMICs, particularly in Latin America, Eastern Europe, and South Asia [12]. Though not specific to vaccines, data show that about 30% of clinical trials sponsored by US-based companies from 1995 to 2005 were carried out in LMICs, without targeting diseases relevant to the host countries, and only 10 out of 1556 newly developed drugs between 1975 and 2004 targeted diseases particularly relevant to host countries [6]. 

This discrepancy is largely due to the fact that the clinical trials carried out in LMICs are sponsored and conducted by companies based in HICs. Between 2002 and 2007, the number of active Food and Drug Administration (FDA)-regulated investigators based outside the US grew by 15% annually, whereas the number of US-based investigators had declined by 5.5%. As of November 2007, one-third of industry-sponsored phase 3 clinical trials for the 20 largest US-based pharmaceutical companies were being conducted solely outside the US, and the majority of study sites were outside the US [13]. Between 2006 to 2013, over a quarter of clinical trials were conducted in non-HICs [14]. and according to data from the FDA and the Department of Health and Human Services, there has been an acceleration in clinical trials for medical products intended for FDA approval being conducted in foreign sites. This has presumably substantially increased since then, though more recent specific data are not readily available. In 2008, 80% of applications for drugs and biologics contained data from non-US-based studies, 78% of all participants were enrolled outside the US, and 8.3% of new drug applications were conducted entirely outside of the US [15].

In theory, locally sponsored clinical trials would better be able to navigate the healthcare landscape, garner trust from communities, and, most importantly, address locally relevant diseases and make accessible affordable interventions for the local populace. However, there are significant barriers to this.

### 3.2. Factors Affecting Clinical Trial Implementation

#### 3.2.1. Regulations

In the US, the FDA has a delicate line to toe. On the one hand, it must be lenient enough to allow for meaningful drug development and delivery in a timely fashion, and, on the other hand, it must be strict enough to ensure the safety of human participants. In times of public health crises, there is often public pressure to expedite approval of drugs and vaccines to limit the number of deaths from life-threatening diseases. This was seen in the 1980s and 90s in the US during the HIV crisis and most recently, worldwide, during the COVID-19 pandemic. Nevertheless, relatively strict regulations in the US, in part, started to lead to the relocation of clinical trials to LMICs in the 1980s [12]. In addition to fewer regulations, the population density in certain LMICs allowed for the recruitment of large numbers of participants, often a bottleneck for research conducted in the US and other HICs. 

While this could potentially allow for more equitable clinical trials, regulations for the protection of study participants can vary greatly between countries and can allow for the exploitation of these populations, many of whom may be of low socioeconomic status within their own countries. In one study, only 56% of the 670 researchers surveyed in developing countries reported that their research had been reviewed by a local IRB or health ministry [13]. There are guidelines in place for the ethical implementation of human subjects research regardless of location, namely, the Declaration of Helsinki (DoH), but it is not a legally binding guideline. A major revision was made to the DoH in 2000 in response to studies performed with US federal funds that denied effective medication to participants in the placebo control arm of a study on HIV transmission. However, these revisions are scarcely referred to when regulating studies outside of the US [12].

In response to poor regulation in LMICs, the World Health Organization (WHO) introduced the Global Benchmarking Tool to evaluate national health regulatory agencies in LMICs in several areas, including regulatory inspection, laboratory testing, and clinical trial oversight. The process is lengthy, taking between 2 and 5 years, but seeks to identify ways in which regulatory programs can be strengthened to effectively conduct clinical trials (among other endeavors). Investment in this effort may allow efficient and equitable implementation of clinical trials in LMICs [16]. 

Not only are regulations for the protection of study participants variable between countries, but the exportation and implementation of regulations from one country to another do not always work. For example, while the DoH mandates that control groups in clinical trials receive the best current treatment available [17], this is not always feasible or a portrayal of real-world implementation. A notable example is the execution of clinical trials for the drug zidovudine in the 1990s in Sub-Saharan Africa where a placebo was used in clinical trials instead of the best available treatment. This raised ethical objections to the trials; however, closer examination revealed a more complex ethical dilemma. How should such situations be navigated when the “best available” treatment is not readily available to patients within a given population and where placebo does not carry a risk beyond that associated with standard practice? This limits the effectiveness of clinical trials in resource-poor settings where the appropriateness and financial feasibility of providing the intervention are not taken into account. It can also lead to exploitation of the local population where drug/vaccine efficacy is tested in populations that would not reasonably be able to use the intervention [17]. Another issue of regulatory variability includes that of consent. While consent conventionally takes the form of a decision made by the participant, this may not be applicable in other cultures where, for example, a tribal chief’s consent or community agreement is paramount [12]. 

#### 3.2.2. Staffing, Infrastructure, and Logistics

While, in theory, recruitment for and initiation of clinical trials may have less regulatory hurdles in LMICs than in HICs, other challenges emerge when seeking to implement trials in these countries. Some of the most commonly cited barriers are the lack of human capacity, financial infrastructure, research environment, and operational barriers and competing demands [6]. As a result, large US and other HIC-based pharmaceutical companies that have large reservoirs of resources are often able to address these barriers and conduct clinical trials in LMICs, whereas local companies that do not have these resources and that cannot compete with more powerful currencies find these barriers difficult to overcome [6]. Furthermore, when companies based in LMICs do engage in clinical trials, there is often a large turnover in staff as there are few people with comparable expertise within communities that rarely host research. These staffing shortages create a bottleneck in the conduct of clinical trials. 

These obstacles were further magnified during the COVID-19 pandemic. Many LMICs did not have the necessary facilities, equipment, and human capacity to conduct research and/or clinical trials [6,9]. So, while vaccine clinical trials for SARS-CoV-2 did include much more diverse human populations than other clinical trials in the past, they were most often conducted by pharmaceutical companies based in HICs. However, as opposed to the development of vaccines for other infectious diseases in the past, countries with emerging economies were also at the forefront of vaccine development for SARS-CoV-2, such as China, Russia, and India. While the economies of these countries and their designations as HICs, MICs, or LICs are beyond the scope of this review, these countries are not representative of much of the developing world. Interestingly, while funding for vaccine development largely comes from private sources (i.e., pharmaceutical companies), unprecedented amounts of funding came from government sources worldwide for the development of a SARS-CoV-2 vaccine, enabling countries with emerging economies to invest in vaccine development [18]. 

#### 3.2.3. Attitudes towards Research

Numerous scandals surrounding medical research have affected public trust in clinical trials. This includes the Tuskegee Syphilis Study in the US and the thalidomide scandal in Germany in the early 1960s. These studies have exposed the dangers of poor regulation surrounding the ethics of medical research [12]. Both studies have left a lasting impression on communities’ trust in medical research. This is a sentiment held in both HICs and LMICs. While this belief may hinder participation in clinical trials, it is still often easier to recruit and enroll patients into clinical trials from LMICs, particularly patients of a lower socioeconomic status or those who perceive involvement in trials as a means to receive access to necessary healthcare they would otherwise be unable to obtain. This poses ethical concerns about consent and the exploitation of participants, particularly when financial compensation for clinical trial participation can exceed a participant’s annual wages [13].

### 3.3. Lessons from Vaccine Clinical Trials on Clinical Trial Access and Real-World Implementation 

#### 3.3.1. SARS-CoV-2

While disparities in access to vaccination itself between HICs and LMICs are well known, vaccine inequity in association with clinical trials is less known. The race to vaccine development during the COVID-19 pandemic was unprecedented. On 10 January 2020, Chinese scientists shared the SARS-CoV-2 sequence, and 63 days later, the first human vaccine clinical trial began in the US. Shortly thereafter, trials were conducted in other HICs and LMICs alike. However, the benefits of these trials were not shared equitably [19]. 

**Figure 1:**
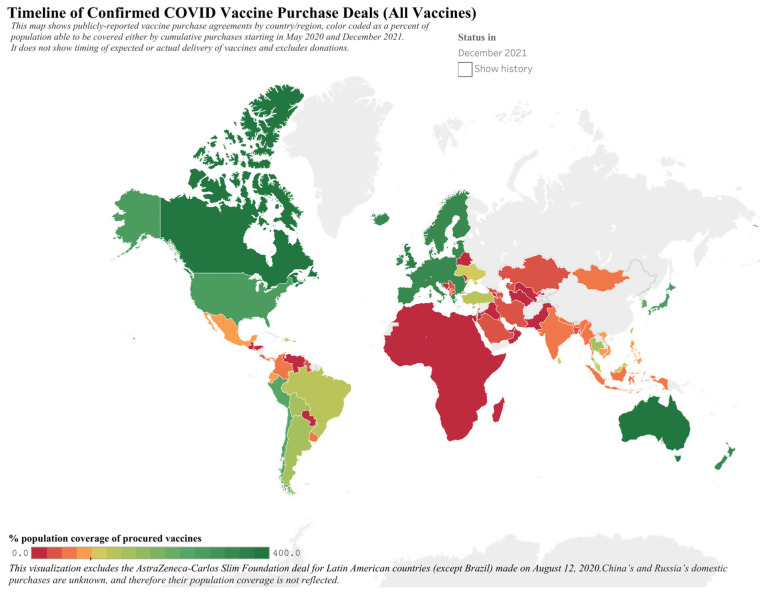
Depiction of COVID/SARS-CoV-2 vaccine purchase agreements by country as of December 2021. Figure re-published under Creative Commons License from https://launchandscalefaster.org/covid-19/vaccinepurchases, accessed on 12 November 2023 [22].

Ramachandran et al. examined whether countries that tested a specific COVID-19 vaccine went on to authorize and have access to that vaccine. Examining ongoing and completed trials, authorization rates for tested vaccines were similar between HICs and LMICs, but actual access to vaccines and vaccination rates were considerably lower for LMICs, enabling HICs to more fully vaccinate their populations [20]. The exact reasons for this disparity are not entirely clear but appear to be related to HICs, and particularly those that funded clinical development of vaccines, entering into agreements with vaccine manufacturers for vaccines for their own populations, and leaving out countries which were not able to engage in such agreements but whose populations may have participated in clinical trials (Table 1) [21]. This hoarding of vaccines by HICs led to immense wastage of vaccines and, because of global manufacturing constraints, also left a smaller number of vaccines available for equity-focused partnerships like the WHO initiative COVID-19 Vaccines Global Access (COVAX). By the end of 2021, HICs were on course to have 1 billion unused doses and enough doses to vaccinate all their citizens three to six times over, while other largely LICs and the entire continent of Africa were unable to provide just the first dose of the vaccine to the vast majority of their populations (Figure 1) [20,22]. And by the end of April 2022, over 81% of vaccine doses had been administered to people residing in high- and upper-middle-income countries, while just 0.3% had been provided to those in LICs, resulting in only 20% of the world’s population receiving 95% of all doses [23].

While further discussion on disparities in access to the COVID-19 vaccine is beyond the scope of this review, it is important to recognize the unique, acute, and global reach and scale of the pandemic in relation to recent history. Other pandemics in recent history did not have the same virulence or global impact. The 2009 swine flu (H1N1) pandemic, for example, resulted in approximately 284,000 deaths worldwide in its first 12 months, whereas the COVID-19 pandemic resulted in approximately 2,000,000 deaths worldwide in its first 12 months [24]. Similarly, the 1957 H2N2 and 1968 H3N2 flu pandemics were each responsible for ~1,000,000 deaths worldwide through the entirety of their respective pandemics [25]. The global scale of the COVID-19 pandemic along with an environment of rapidly evolving technology paved the way for an unprecedented level of international coordination and clinical trial execution and dictated a massive global need for acute access to vaccination. 

While outcomes of these clinical trials and rapid vaccine development highlight the benefits of global cooperation and inclusion of LMICs, it also exposes the deficiencies and disparities that exist, particularly in equitable access to vaccines after clinical trials, and the lack of local clinical trial initiatives in LMICs, undermining their participation as stakeholders in their own trajectories. 

#### 3.3.2. Poliovirus

The poliomyelitis epidemic is a prime example of the need to tailor vaccines to specific populations and the value of local clinical trials. Poliomyelitis has existed for thousands of years, but the virus that causes it was first identified in 1909. Outbreaks in Europe and the US in the late 19th century spurred the development of polio vaccines, and the first successful polio vaccine was created by Jonas Salk in the 1950s. Salk first tested the inactivated polio vaccine (IPV) on himself and his family and, a year later, on 1.6 million children in Canada, Finland, and the US. In 1955, the IPV was licensed, and within 2 years, there was a dramatic drop in polio in the US. However, this could not be replicated in other parts of the world, particularly in LMICs. In most HICs and upper MICs, the IPV is administered as part of national vaccination programs [26]. However, the IPV is expensive, and many LMICs cannot afford mass immunization campaigns. An alternative to the IPV, a live attenuated oral polio vaccine (OPV), was also developed in the 1950s by researchers in the US, but given the success of the IPV, there was little interest in pursuing testing of this option. While the IPV had a favorable risk–benefit profile in HICs, the presence of poliovirus in any country was a global threat, and burgeoning interest internationally led to the testing of the OPV in Belgian Congo, the Soviet Union, and Czechoslovakia in the 1950s and 1960s [27]. 

The OPV does have risks associated with it, including the risk of vaccine-associated paralytic poliomyelitis and the generation of circulating vaccine-derived poliovirus (cVDPV). In LMICs, fractional doses of the IPV are often used in conjunction with the OPV, to lessen the financial impact of IPV immunization [28]. Local clinical trials have demonstrated that although fractional doses result in lower seroconversion rates, they effectively boost pre-existing antibody titers. Furthermore, a trial in Sri Lanka demonstrated that in children with a previous OPV dose, fractional doses of IPV boost mucosal immunity similar to that of a full dose of IPV [29]. In countries where polio remains endemic (i.e., Afghanistan and Pakistan), the OPV is preferred as immunity can be conferred between individuals. This is of particular benefit in regions where cultural, social, and educational barriers to vaccination may affect vaccination campaigns, albeit at the risk of the development of cVDPV when there is low vaccination coverage in the general population.

The differential feasibility of the polio vaccine speaks to the need for locally relevant implementation strategies, and clinical trials to test them, for effective control of infectious disease.

#### 3.3.3. Malaria

Malaria is a parasitic disease with many unique barriers to the development of a vaccine. In 2020, there were 241 million cases of malaria and 627,000 resulting deaths, most of them among children younger than 5 years of age in Sub-Saharan Africa [30]. While several efforts to develop a vaccine against malaria and test distribution and implementation strategies are underway, the development of a malaria vaccine has faced unique obstacles including the lack of a traditional market, few developers, and poor understanding of the complex immune response to malaria infection [31]. 

**Figure 2:**
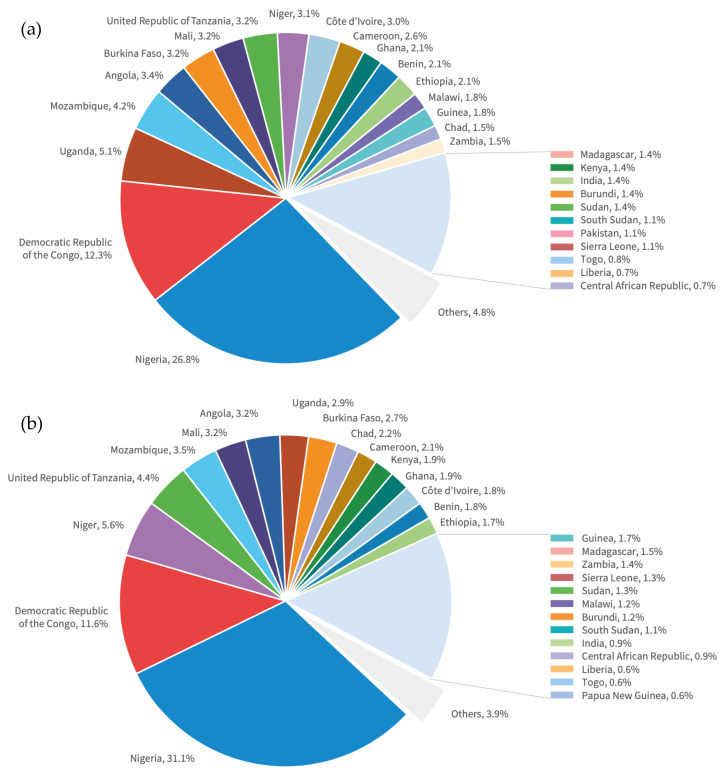
Global trends in (**a**) distribution of malaria cases and (**b**) deaths by country in 2022. Adapted and published under Creative Commons License from World Malaria Report, accessed on 14 February 2024 [32].

In regions where malaria is endemic, the disease disproportionately affects the poorest sectors of society. Malaria is both a consequence and a cause of poverty, and inequality and the lack of an effective vaccine amplifies this [30]. Almost half of all cases were accounted for by four countries in Sub-Saharan Africa (Nigeria, the Democratic Republic of the Congo, Uganda, and Mozambique), and four countries accounted for just over half of all malaria deaths globally (Nigeria, the Democratic Republic of the Congo, Niger, and the United Republic of Tanzania) [32]. See Figure 2.

While vaccine clinical trials are a necessary component in addressing the prevention of malaria and mortality, questions of end-of-trial obligations from researchers and pharmaceutical companies to the local populations on whom vaccines are tested arise. A recent malaria vaccine trial is a unique case study in this regard. A phase II/III pediatric malaria vaccine trial (PMVT) in Ghana and Tanzania serves as a case example of analyzing the public health impact that conducting non-locally initiated research may have on healthcare infrastructure.

Ward et al. explored stakeholder experiences and perspectives on end-of-trial obligations at the close of the PMVT [33]. These perspectives were shared on the themes of communicating the end of the trial, maintaining healthcare service, dissemination of results, and post-trial availability. They noted that although the participants knew the project was time-limited, the change in access to healthcare after the completion of a trial created anxiety and feelings of abandonment in the community. And while all respondents recognized the positive infrastructural changes to the local health system the trial brought, there were concerns over the cost of maintaining the availability of these resources. Lastly, and perhaps most challenging, was the concept of having to make post-trial commitments of vaccine availability in the context of a single clinical trial as this alone does not usually lead to an immediate intervention that can be implemented in a population. A significant amount of testing and regulatory hurdles must be met before a vaccine can be brought to market and licensed for general access. 

### 3.4. Perspectives from LMICs on Locally Led Clinical Trials 

Few studies have documented the perspectives of LMICs on local clinical trial implementation and research infrastructure [34]. In 2013, the World Health Report stated that “all nations should be producers and users of research”, and in 2005, the WHO made a statement on making the establishment of African-owned research centers capable of running their own clinical trials an international priority. Despite some progress, the vast majority of research is still led by HICs, and many LMICs cannot sufficiently undertake research, specifically clinical trials [6,35]. A systematic review of the health research capacity development literature found the development of locally led trial capacity in LMICs has been largely ignored. It identified only three papers that were dedicated to considering how locally led trial capacity could be developed, and none of these were empirical. Perspectives on clinical trial conduct of stakeholders on clinical trials in LMICs themselves are poorly documented in the literature. As a result, Franzen et al. conducted a prospective case study consisting of interviews, focus group discussions, and process mapping exercises with local health researchers in the LMICs of Ethiopia, Cameroon, and Sri Lanka, to identify barriers to locally led clinical trial conduct in LMICs and determine strategies for their sustainable development. Common barriers included themes within stewardship and governance, financing, creating and sustaining resources, and producing and using research. Specifically, inefficient governance, weak research stewardship, bureaucracy, and weak regulatory frameworks preclude the conduct of clinical trials. In addition, there is little priority given to research and related finances, with the focus of universities being teaching and not research. Lastly, limited material capacity, lack of human capacity to conduct research, lack of awareness of and exposure to clinical trial conduct, and low motivation to conduct research with few career incentives or recognition were cited as barriers to locally led trial initiatives. Franzen et al. went on to describe strategies for developing sustainable health research capacity in LMICs and commented on the need to foster proresearch cultures, develop trial leaders and staff, provide a facilitative operational environment, and ensure that trial research has a local impact.

## 4. Discussion

In the last several decades, there has been much debate as to the benefits and detriments of the globalization of clinical trials. Vaccine clinical trials are of particular interest to prevent the spread of infectious diseases. In an increasingly interconnected world, infectious diseases in one corner of the earth can affect the entire world, as was seen with the COVID-19 pandemic. While the COVID-19 pandemic did see an increase in global participation in vaccine clinical trials, the disparities between HICs and LMICs were still stark. Most vaccine clinical trials for COVID-19 still took place in HICs with only 25 of 219 trials (11.4%) taking place in LMICs (the two LICs were Guinea-Bissau and Kenya) with an extreme paucity of clinical trials being conducted in central and south-east Asia and the entire continent of Africa. These disparities in clinical trial representation affect the external validity and applicability of vaccines as geographical, infrastructural, and racial differences are not accounted for when vaccines are primarily or solely tested in HICs [36]. 

While a global pandemic presents the opportunity to include LMICs in industry-funded clinical trials, even where clinical trials for SARS-CoV-2 vaccines were conducted in LMICs, there were concerns of significant inequities in the benefits drawn from these trials in HICs versus LMICs. Additionally, pandemics are relatively rare, and most infectious diseases are locally confined or regional epidemics. This creates a scenario where populations in LMICs can be particularly exploited because they can be used by large pharmaceutical companies based in HICs to test vaccines for diseases that do not affect the local population. Furthermore, advancement in diseases that do affect LMICs stagnates as pharmaceutical companies have little vested interest in relevant vaccine development, and local infrastructure cannot support the development or testing of vaccine candidates.

While many barriers exist on the road to implementation, the most viable solution for equitable inclusion of LMICs in vaccine clinical trials appears to be enabling these countries to conduct their own locally relevant trials for diseases that are a local health burden and to make their own medical products [17]. Not only can vaccine effectiveness for their populations be more accurately assessed, but the storage, infrastructure, and delivery strategies necessary to effectively distribute vaccines and implement findings from the clinical trials can be applied to the country’s specific settings and needs. Cold chain storage and multi-dose injections, for example, were requirements that proved troublesome in LMICs in the storage and delivery of SARS-CoV-2 vaccines [36]. There was also public outcry when Johnson & Johnson exported COVID-19 vaccine doses made in South Africa to already heavily vaccinated European countries [19]. Furthermore, the positive impact on health and social systems that clinical trials can effect, such as strengthened physical infrastructure, better-trained staff, and improved community health-seeking behaviors, can only be sustainable for LMICs when they are their own stakeholders [33]. Lastly, while ethics in clinical research must be closely observed regardless of region, certain principles are universal, while others may not be. With internally initiated clinical trials in LMICs, ethical considerations in the implementation and conduct of clinical trials can be examined in culturally and socially relevant ways. 

While large emerging economies like those of China, India, Russia, and Brazil have developed, or are rapidly developing, the infrastructure needed to conduct their own clinical trials and, therefore, tailor implementation in a manner that is unique and most effective for their populations, the vast majority of LMICs do not currently have this capability, and the largest bottleneck may be a lack of human resources. In addition to government and locally financed clinical trials in LMICs, highly qualified personnel are needed to propose, initiate, and conduct clinical trials from within the country [6]. A well-established medical and public health infrastructure is needed in order to cultivate this human resource, without which the feasibility of clinical trials is poor [36]. Economic constraints in LMICs mean resource and funding allocation for research development is limited to nonexistent. While programs such as the WHO Global Benchmarking Tool may be the beginning of one avenue to the local execution of vaccine clinical trials in LMICs, it does not address the lack of funding allocation for clinical trial development [37]. Other measures, such as the European & Developing Countries Clinical Trials Partnership and the Global Health Trials Network, which aim to provide free resources for career development for clinical trial professionals and clinical trial capacity building, do not address the underlying obstacles to establishing the medical and public health infrastructure needed [38]. 

Case examples from vaccine clinical trials for SARS-CoV-2, poliovirus, and malaria highlight the unique real-world considerations of clinical trials in LMICs. The COVID-19 pandemic, in particular, demonstrated the power of international cooperation and competition, but also showcased the stark inequities in clinical trial access and implementation and subsequent inequitable vaccine distribution even among populations in which the vaccine was tested in clinical trial settings. Diseases, such as malaria, that are endemic to the poorest regions of the world are also left behind due to lack of a lucrative market. And regional economic and storage considerations regarding vaccines can reduce real-world applicability of vaccine clinical trial outcomes as was seen with poliovirus. 

How the international community and governmental and non-governmental organizations can make a concerted effort to prioritize the use of scarce resources and increase funding for the establishment of research infrastructure in LMICs so that they can conduct their own locally relevant and powered clinical trials remains to be seen. Only when this is accomplished can LMICs become active stakeholders in the health of their citizens (as it pertains to vaccine research and development) by addressing endemic diseases, tailoring vaccine specifications based on local needs, and implementing wide-scale vaccine access and delivery.

## Figures and Tables

**Table 1:** Access to any COVID-19 vaccine in countries with completed COVID-19 vaccine clinical trials categorized by national income group (as of 7 September 2021). Figure adapted and re-published under Creative Commons License from Ramachandran et al. [20].

	No. (%)			Proportion of Population Fully Vaccinated with Doses Delivered of Tested Vaccine, Median (IQR), %
	Countries that Hosted COVID-19 Vaccine Clinical Trials	Countries that Authorized Tested Vaccine	Countries with Doses Delivered of Tested Vaccine	
Completed clinical trials				
All	25	23 (92.0)	23 (92.0)	39.4 (14.3–74.7)
Low income	0	NA	NA	NA
Lower middle income	3 (12.0)	3 (100.0)	3 (100.0)	31.0 (18.1–37.6)
Upper middle income	11 (44.0)	10 (90.9)	10 (90.9)	14.9 (7.2–48.6)
High income	11 (44.0)	10 (90.9)	10 (90.9)	51.7 (39.4–76.7)
Ongoing and completed clinical trials				
All	37	35 (94.6)	35 (94.6)	39.5 (14.3–74.7)
Low income	1 (2.7)	1 (100.0)	1 (100.0)	6.4
Lower middle income	6 (16.2)	6 (100.0)	6 (100.0)	20.2 (9.3–29.0)
Upper middle income	12 (32.4)	10 (90.9)	10 (83.3)	14.6 (1.6–55.4)
High income	18 (48.6)	18 (100.0)	18 (100.0)	67.9 (44.3–78.9)

Abbreviations: COVAX, COVID-19 Vaccines Global Access; NA, not applicable.

## Data Availability

No new data were created or analyzed in this study. Data sharing is not applicable to this article.

## Appendix A

PubMed Search Strategy:

(“Vaccines”[MeSH Major Topic] OR “Vaccine Development”[MeSH Terms] OR vaccin*[Title/Abstract]) AND (“Clinical Trials as Topic”[MeSH Terms] OR trial*[Title/Abstract] OR vaccine trial*[Title/Abstract] OR vaccine test*[Title/Abstract]) AND (“Health Services Accessibility”[MeSH Terms] OR “Healthcare Disparities”[MeSH Terms] OR inequit*[Title/Abstract] OR inequalit*[Title/Abstract] OR equit*[Title/Abstract] OR equalit*[Title/Abstract] OR disparit*[Title/Abstract] OR “Health Services Availability”[Title/Abstract] OR “Access To Care, Health”[Title/Abstract] OR “Access to Health Care”[Title/Abstract] OR “Program Accessibility”[Title/Abstract] OR “Accessibility, Program”[Title/Abstract] OR “Health Services Geographic Accessibility”[Title/Abstract] OR “Access to Therapy”[Title/Abstract] OR “Access to Therapies”[Title/Abstract] OR “Therapy, Access to”[Title/Abstract] OR “Access to Treatment”[Title/Abstract] OR “Access to Treatments”[Title/Abstract] OR “Treatment, Access to”[Title/Abstract] OR “Access to Medicines”[Title/Abstract] OR “Access to Medicine”[Title/Abstract] OR “Medicine, Access to”[Title/Abstract] OR “Medicines, Access to”[Title/Abstract] OR “Access to Medications”[Title/Abstract] OR “Access to Medication”[Title/Abstract] OR “Medication, Access to”[Title/Abstract] OR “Medication Access”[Title/Abstract] OR “Access, Medication”[Title/Abstract]) AND (“Developing Countries”[MeSH] OR Developing countr* OR low income countr* OR Third-World* OR Third World* OR under-developed* OR Under developed* OR LMIC* OR Least Developed Countr* OR Least-developed countr*)
